# Density of *Aedes aegypti* (Diptera: Culicidae) in a low-income Brazilian urban community where dengue, Zika, and chikungunya viruses co-circulate

**DOI:** 10.1186/s13071-023-05766-5

**Published:** 2023-05-06

**Authors:** Raquel L. Souza, Romero J. Nazare, Hernan D. Argibay, Maysa Pellizzaro, Rosângela O. Anjos, Moyra M. Portilho, Leile Camila Jacob-Nascimento, Mitermayer G. Reis, Uriel D. Kitron, Guilherme S. Ribeiro

**Affiliations:** 1 Instituto Gonçalo Moniz, Fundação Oswaldo Cruz, Salvador, Bahia Brazil; 2 Universidade Federal da Bahia, Salvador, Bahia Brazil; 3 Yale School of Public Health, New Haven, CT USA; 4 Emory University, Atlanta, GA USA

**Keywords:** *Aedes aegypti*, Environmental monitoring, Environmental indicators, Socioeconomic factors, Mosquito-borne diseases, Mosquito vectors, Mosquito control, Water supply

## Abstract

**Background:**

Low-income urban communities in the tropics often lack sanitary infrastructure and are overcrowded, favoring *Aedes aegypti* proliferation and arboviral transmission. However, as *Ae. aegypti* density is not spatially homogeneous, understanding the role of specific environmental characteristics in determining vector distribution is critical for planning control interventions. The objectives of this study were to identify the main habitat types for *Ae. Aegypti*, assess their spatial densities to identify major hotspots of arbovirus transmission over time and investigate underlying factors in a low-income urban community in Salvador, Brazil. We also tested the field-collected mosquitoes for arboviruses*.*

**Methods:**

A series of four entomological and socio-environmental surveys was conducted in a random sample of 149 households and their surroundings between September 2019 and April 2021. The surveys included searching for potential breeding sites (water-containing habitats) and for *Ae. aegypti* immatures in them, capturing adult mosquitoes and installing ovitraps. The spatial distribution of *Ae. aegypti* density indices were plotted using kernel density-ratio maps, and the spatial autocorrelation was assessed for each index. Visual differences on the spatial distribution of the *Ae. aegypti* hotspots were compared over time. The association of entomological findings with socio-ecological characteristics was examined. Pools of female *Ae. aegypti* were tested for dengue, Zika and chikungunya virus infection.

**Results:**

Overall, 316 potential breeding sites were found within the study households and 186 in the surrounding public spaces. Of these, 18 (5.7%) and 7 (3.7%) harbored a total of 595 and 283 *Ae. aegypti* immatures, respectively. The most productive breeding sites were water storage containers within the households and puddles and waste materials in public areas. Potential breeding sites without cover, surrounded by vegetation and containing organic matter were significantly associated with the presence of immatures, as were households that had water storage containers. None of the entomological indices, whether based on immatures, eggs or adults, detected a consistent pattern of vector clustering in the same areas over time. All the mosquito pools were negative for the tested arboviruses.

**Conclusions:**

This low-income community displayed high diversity of *Ae. aegypti* habitats and a high degree of heterogeneity of vector abundance in both space and time, a scenario that likely reflects other low-income communities. Improving basic sanitation in low-income urban communities through the regular water supply, proper management of solid wastes and drainage may reduce water storage and the formation of puddles, minimizing opportunities for *Ae. aegypti* proliferation in such settings.

**Graphical Abstract:**

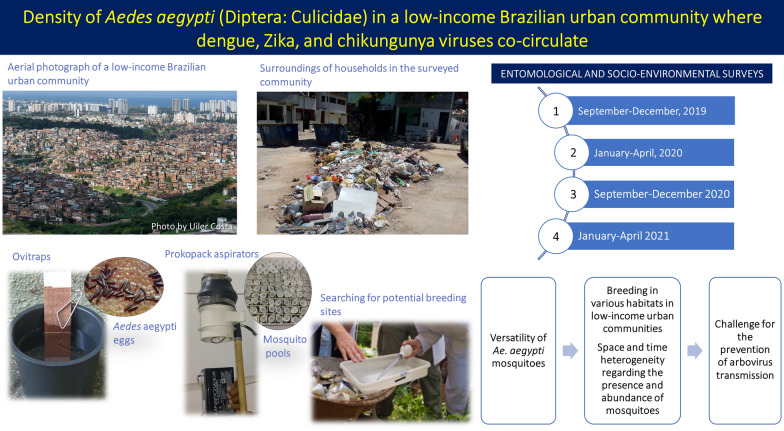

**Supplementary Information:**

The online version contains supplementary material available at 10.1186/s13071-023-05766-5.

## Background

The public health burden of arboviral infections has increased in recent decades, largely associated with the co-circulation of dengue (DENV), Zika (ZIKV) and chikungunya (CHIKV) viruses in the Americas [1, 2] and the continued failure of vector control programs [3, 4]. One reason for the lack of success of vector control strategies is the adaptation of *Aedes aegypti*, the primary vector of DENV, ZIKV and CHIKV, to the urban habitat and its preference to breed in artificial water containers [5, 6]. Furthermore, the urban environment can be diverse, comprising within the same city areas with adequate housing, sanitation and services, and areas without those features, marked by poverty. The latter, generally designated as slums or vulnerable communities (*comunidades*) in Brazil, have a higher risk of arboviral epidemics because their inhabitants live in crowded conditions, facilitating multiple interactions between the vector and humans and because their environment offers more opportunities for *Ae. aegypti* reproduction [7–11].

Although several studies have shown that vulnerable urban areas have higher levels of *Ae. aegypti* density, such studies are often based on household surveys of the immature forms of the vector; investigating the presence of mosquito eggs and adults has been less common [12, 13]. Surveying for both oviposition and adult mosquitoes is a gap in such studies since eggs and adults may be better indicators of the presence of the vectors at a stage capable of arbovirus transmission. In addition, the infestation indices produced from these inspections are often derived from qualitative measures (i.e., presence vs. absence of immature per house), which do not consider mosquito density. Typically, they also do not include data from public areas, which have been shown to harbor important habitats for *Ae. aegypti* reproduction [14–16]. Finally, the coarse-resolution study designs, in which vector data are aggregated over relatively large geographical zones, such as neighborhoods or census tracts, may hamper the detection of localized foci of vector infestation, especially inside vulnerable communities.

Studies that use non-aggregated data to determine different entomological indices and their relations with specific ecological characteristics within low-income urban communities may help detect and assess the importance of vector infestation hotspots and inform on the role of socio-environmental deficiencies in the distribution of *Ae. aegypti*. Despite differences between low-income communities, they share similarities, such as poverty, inadequate housing and sanitation, and high population density. Thus, some findings from one community may be generalizable to others.

In this study, a series of entomological and socio-environmental surveys was carried out in a low-income Brazilian urban community using diverse vector capture methods. The study’s main aims were to investigate whether specific environmental factors were associated with the detection of *Ae. aegypti* mosquitoes, how heterogeneous the vector distribution was within the community and whether foci of *Ae. aegypti* infestation in the community persisted over time. We also evaluated whether the collected specimens of *Ae. aegypti* were infected by DENV, ZIKV or CHIKV.

## Methods

### Study design

Four entomological and socio-environmental survey cycles were conducted in the community of Pau da Lima, Salvador, Brazil, between September 2019 and April 2021 (cycle 1: September–December 2019, cycle 2: January–April 2020, cycle 3: September–December 2020, cycle 4: January–April 2021). We planned to conduct an additional survey during May–Aug 2020; however, due to the COVID-19 pandemic and the restrictions on field activities, we had to cancel it. These cycle periods (January–April, May–August, September–December) were defined based on the rainfall and insolation data for the last 10 years in Salvador [17], which indicated an increase in precipitation and a decrease in insolation between May and August, an inverse pattern from September to December and an intermediate climatic pattern of less precipitation and highest insolation between January and April.

The Research Ethics Committee of Gonçalo Moniz Institute, Oswaldo Cruz Foundation (CAAE: 57221816.8.0000.0040), approved the study, and an adult (≥ 18 years old) who was responsible for the household signed the informed consent term before we performed the survey at their home.

### Study site and selection of households

**Fig. 1:**
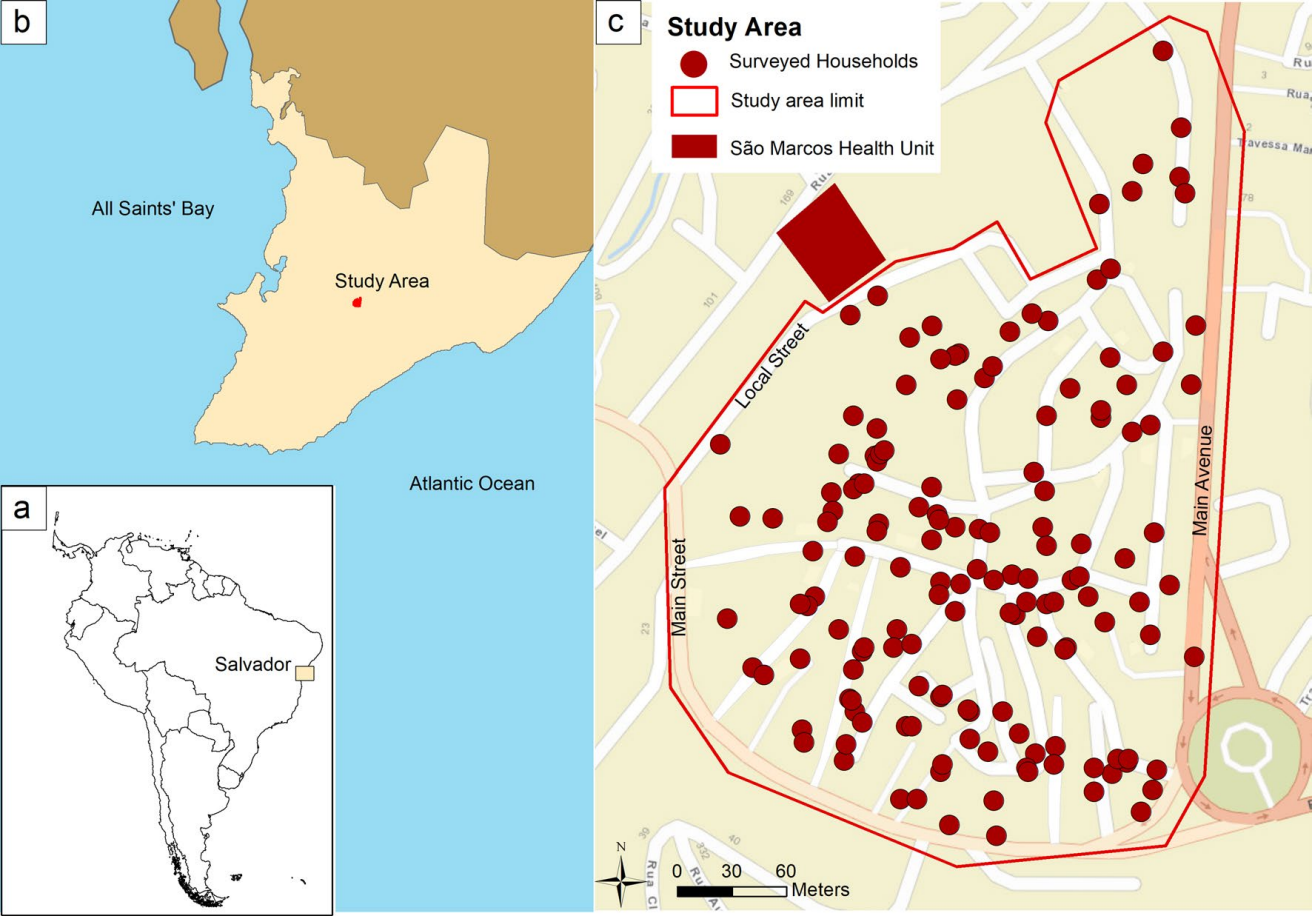
Study area in the Pau da Lima neighborhood, Salvador, Bahia, Brazil. **a** Location of Salvador in Brazil. **b** Location of the study area in Salvador. **c** Study area showing the 149 surveyed households. Due to loss of follow-up, not all 149 households inspected during the first survey cycle were inspected during the subsequent survey cycles

The Pau da Lima community harbors a highly dense low-income population, which lacks basic sanitation conditions [7, 18]. The study site within Pau da Lima was defined based on geographical boundaries delimited by a main avenue, a main street and a local access street (area of 0.082 km^2^) (Fig. 1). It is located near (< 1 km) the São Marcos Health Unit, an emergency unit where we have conducted surveillance for arboviral infection among patients with febrile illness and detected co-circulation of DENV, ZIKV and CHIKV [7, 18]. In July 2019, a household census at the study site was conducted, and 1566 households were counted. Data on the number of inhabitants (2317) were available for 812 (51.9%) households. Based on the counted households and the estimated number of habitants per household, the study site had a population density of ~ 54,500 habitants/km^2^.

Based on the findings of a pilot study in another neighborhood of Salvador showing that 4.3% of the surveyed households had *Ae. aegypti* immatures in reservoirs with standing water, it was planned to include about 150 of the 1566 households in this study, which would provide 3% precision with 95% confidence for the same expected frequency of households with *Ae. aegypti* immatures. Due to the risk of refusal to participate and loss to follow-up, 200 households within the study site were randomly selected, and the first survey cycle was completed in 149 of them. These households became the target group for the subsequent surveys; the remaining 51 households were not surveyed because their owner refused to participate or because nobody was found at home after at least five attempts. The spatial coordinates of the surveyed households indicated that they were dispersed over the study site (Fig. 1). As *Ae. aegypti* mosquitoes can also reproduce in standing water located in public areas, in addition to the enrolled households (herein defined as the private area surveyed), entomological and environmental surveys were also performed in the public spaces within the study site, including all public structures (vacant public lots, squares, streets and rainwater drainage structures, such as storm drains).

### Overview of entomological and environmental surveys

The surveys consisted of (i) identification and characterization of potential breeding sites (defined as any natural structure or artificial reservoir accumulating water, whether covered or not (except capped bottles) which might serve for mosquito reproduction); (ii) collection of immature forms (larvae and pupae) from breeding sites; (iii) capture of adult mosquitoes by aspiration (only in the private area and in storm drains); (iv) installation of ovitraps (only in the private area, due to the high risk of loss in the public area). Additional details about these strategies are provided below. In addition, during the private area surveys, an adult responsible for the household was interviewed about the frequency of water supply and use of water storage containers (water reservoirs) in the house. The inspections were performed two to three days per week during each survey cycle, from 8:30 a.m. to 2:00 p.m. (average of 8 weeks of survey per cycle).

### Identification and characterization of potential breeding sites

In each survey, systematic inspections searching potential breeding sites were conducted inside and outside all studied households and in all public structures. The spatial coordinates of all the potential breeding sites in the public area and those of the studied households were recorded. They were further characterized by type (water tank, vat, tires, etc.), location (indoor or outdoor, for those found in the households), presence or absence of surrounding plants or trees, presence or absence of cover/protection, estimated water volume, visual presence or absence of organic matter in the water, and presence or absence of immature forms of mosquitoes in the water. During each survey, residents were instructed on mechanical procedures to avoid nonintentional water accumulation in their households and to properly close any water storage containers to reduce arbovirus transmission risk. Whenever possible, the potential breeding sites found during the surveys were eliminated. The detected storm drains were inspected to verify the shading status, measured to estimate the volume of water inside and evaluated for the presence of inorganic matter in the water.

### Collection of immature forms from breeding sites

All the water from small and movable breeding sites (< 3 l) was placed in a white tray to collect all the immature mosquito forms with a pipette. For fixed and relatively large breeding sites (> 3 l) or storm drains, we passed a larvae net ten times in the water to catch immature forms. The immature mosquitoes collected from each breeding site were placed in individual Falcon tubes containing 70% alcohol, which were labeled and taken to the Laboratory of Entomology, located at Oswaldo Cruz Foundation (FIOCRUZ), Salvador, Bahia, Brazil. The immature specimens collected were classified into larva or pupa, identified to the lowest possible identification level using the identification keys in Consoli and Oliveira [19], and quantified.

### Collection of adult forms from households and storm drains

Prokopack aspirators [20] were used to collect adult mosquitoes inside all selected households and in storm drains located in the public area. Aspirations were performed for approximately 10 min inside the homes and 1 min in storm drains. The mosquitoes captured in each household or storm drain were kept in individual closed cups and transported on CO_2_ ice to the Laboratory of Entomology. Two researchers jointly identified the collected specimens to the finest possible identification level using the identification keys described by Consoli and Oliveira [19]. Mosquitoes were grouped into pools of 1–20 specimens (by species, date of collection, capture location site, sex and engorgement status when female) and stored in freezers at – 80 ºC to be further tested for arboviral infections.

### Ovitrap surveys

In each survey cycle, one ovitrap per household was installed (preferably outdoor). The ovitraps, consisting of a black plastic cup filled with about 350 ml of tap water and an oviposition pallet, were removed after 5–7 days. The pallets were placed in individual plastic Ziplock bags, which were labeled and transported to the Laboratory of Entomology, where they were examined under a stereomicroscope (Nikon SMZ 745 T) to count *Ae. aegypti* eggs. When immatures were found in the ovitrap water, they were placed in individually labeled Falcon tubes containing 70% alcohol and brought to the Laboratory of Entomology to be identified as described before.

### Investigation of arboviral infection in mosquitoes

All available pools of adult female *Ae. aegypti* mosquitoes were tested for DENV, ZIKV and CHIKV by qRT-PCR. These comprised 30 pools of non-engorged mosquitoes (a total of 35 mosquitoes) and 34 pools of engorged mosquitoes (a total of 40 mosquitoes). Pools were macerated using a Tissue Lyser L-Beader 6 (Loccus, São Paulo, Brazil) containing one zirconia bead and 500 µl fetal bovine serum and then centrifuged for 10 min at 10,000 ×*g* at 4 °C. According to the manufacturer's protocol, the supernatant was used to extract RNA with the Maxwell^®^ 16 Viral Total Nucleic Acid Purification kit (Promega, Wisconsin, USA). Extraction products were stored at − 80 °C until tested by real-time qRT-PCR in an ABI Prism 7500 SDS Real-Time cycler (Applied Biosystems, Foster City, CA, USA) using the CDC TRIOPLEX real-time qRT-PCR protocol [21]. Positive and negative controls were included in all extraction rounds and qRT-PCR experiments.

### Data analysis

Data were stored on RedCap (Research Electronic Data Capture, Vanderbilt University, Nashville, TN, USA) [22]. Statistical analyses were performed using STATA 12 statistical software (Stata Corp LP, College Station, TX, USA) [23]. For the spatial analyses, the ArcGis 10.2.2 geoprocessing program was used (Esri, Redlands, CA, USA) and the GeoDa program (Geographic Data Analysis–University of Chicago, IL, USA) [24].

Six infestation indices of *Ae. aegypti* were calculated overall and for each survey cycle as follows [25]. Container Index (CI): percentage of inspected containers positive for the presence of *Ae. aegypti* immature forms; House Index (HI): percentage of inspected households positive for the existence of *Ae. aegypti* immature forms; Breteau Index (BI): the ratio between the number of water containers positive for *Ae. aegypti* immature forms and the number of inspected households, times 100; Ovitrap Positivity Index (OPI): percentage of ovitraps positive for eggs; Egg Density Index (EDI): average number of eggs per positive trap; Adults Index (AI): percentage of households positive for *Ae. aegypti* adults.

The daily averages of temperature and humidity were calculated for the days of field activities through several measurements obtained with a digital thermohygrometer during the georeferencing of the breeding sites located in the public area and during home inspections. Each survey cycle's mean temperature and humidity were then estimated based on daily averages. Pluviometric data were obtained from Salvador’s central meteorological station [17], and the daily rainfall data for the period between the start and end of each survey cycle were used to calculate the average daily levels of rainfall for each survey cycle.

Entomological indices were calculated for the private and public areas for the overall study period and separately for the four survey cycles. The Chi-square test or Fisher's exact test was used to investigate the association between specific characteristics of the potential breeding sites (such as being covered or surrounded by plants) with the presence of immature forms of *Ae. aegypti* inside it, as well as the association of household entomological indicators (existence of eggs, immatures and adults) with household social and environmental characteristics, such as reported regularity of water supply (yes or no), presence of water storage container (yes or no), height (ground or above ground), number of inhabitants within the household (≤ 5 or > 5), type of street pavement (paved or not) and type of residence construction (with or without plastered walls). The level of statistical significance was set at 5%.

Finally, kernel density ratios were used to represent the spatial distribution of the *Ae. aegypti* density indices overall and for each survey cycle. The denominator for the density-ratio calculations were the surveyed households for the immature index, the aspirated households for the adult index and those with recovered ovitraps for the egg index. Univariate Local Moran's I test was used to investigate the existence of spatial clustering for each of the *Ae. aegypti* indices (eggs, immatures, and adults) overall and for each survey cycle.

## Results

### Detection of potential *Ae. aegypti* breeding sites

During the first survey cycle, 149 households were inspected. In subsequent cycles, the number of households examined decreased because of loss to follow-up (moving of residents of the selected households, refusals or inability to find someone who could receive our team); 112, 98 and 89 households were inspected in each of the following three surveys in the private area for a total of 448 household inspections during the study period (Additional file 1).

Overall, 316 water containers or structures containing water with the potential to serve for *Ae. aegypti* reproduction were found in the private area during the four survey cycles. This represents 70.5 potential breeding sites per 100 inspected households during the four survey cycles. Despite the high frequency of potential breeding sites in the private area (316), *Ae. aegypti* immature forms were only found in 18 of them (Table 1). Thus, the relative frequency of breeding sites containing *Ae. aegypti* immature forms in the private area (CI) was 5.7%. The relative frequency of households with *Ae. aegypti* immature forms (HI) was 4.0% (18 out of 448 inspected households) (Table 1). Because all households where we found water containers with immature forms of *Ae. Aegypti* only had one water container positive for the presence of *Ae. Aegypti*, the HI and BI were the same during the study.

**Table 1:** Frequency of entomological indices for the private and public areas for the overall study period and for the four survey cycles separately, Pau da Lima neighborhood, Salvador, Brazil, September 2019 to April 2021

Entomological index, according to the type of area	Survey cycle				
	Overall	1	2	3	4
Private area					
Number of inspected households^a^	448	149	112	98	89
Number of potential breeding sites	316	107	111	67	31
Frequency of potential breeding sites per 100 households	70.5	71.8	99.1	68.3	34.8
Number of potential breeding sites positive for *Aedes aegypti* immatures	18	5	6	5	2
Frequency of potential breeding sites positive for *Ae. aegypti* immatures, in percentage (container index)	5.7	4.6	5.4	7.4	6.4
House index, percentage	4.0	3.3	5.3	5.1	2.2
Breteau index, percentage	4.0	3.3	5.3	5.1	2.2
Total number of *Ae. aegypti* immatures in breeding sites	595	204	269	55	67
Average number of *Ae. aegypti* immatures per positive breeding site	33	40.8	44.8	11.0	33.5
Number of recovered ovitraps	373	126	90	85	72
Number of recovered ovitraps with eggs	157	60	33	35	29
Ovitrap Positivity Index, percentage	42.1	47.6	36.6	41.1	40.2
Number of eggs in the ovitrap pallets	7299	4075	835	1681	708
Egg Density Index, average	46.5	68.0	25.3	48.0	24.4
Number of households undergoing mosquito aspiration	446	147	112	98	89
Number of households with *Ae. aegypti* adults captured	81	19	23	22	17
Adult Index, percentage	18.2	12.9	20.5	22.4	19.1
Number of *Ae. aegypti* adults captured	138	27	41	36	34
Public area					
Number of potential breeding sites	186	89	19	58	20
Number of potential breeding sites positive for *Ae. aegypti* immatures	7	3	1	2	1
Frequency of potential breeding sites positive for *Ae. aegypti* immatures, percentage (container index)	3.7	3.3	5.2	3.4	5.0
Total number of *Ae. aegypti* immatures in breeding sites	283	29	138	115	1
Average number of *Ae. aegypti* immatures per positive breeding site	40.4	9.6	138.0	57.5	1.0

Potential breeding sites were defined as any structure or reservoir accumulating water

^a^The number of households inspected decreased during follow-up because there was a loss of follow-up in relation to the initial sample of 149 households

During the four survey cycles, 186 structures or reservoirs containing water with the potential to serve for *Ae. aegypti* reproduction were found in the public area, of which 7 (3.7%) had immature *Ae. aegypti* (Table 1). This CI (3.7%) was not statistically different from that observed in the private area (5.7%) (*P* = 0.33). Notably, the study area only had one storm drain, which was dry in all four survey cycles; therefore, no mosquitoes (immature or adult) were collected.

### Quantification of immature forms of *Ae. aegypti* by breeding site type

The 18 water containers with immatures of *Ae. aegypti* found in the private area harbored 595 immature *Ae. aegypti*. Among the types of water containers identified in the private area during the four surveys, the most productive for *Ae. aegypti* were water buckets, water tanks (connected to the water supply service) and plastic containers, which contributed to 393 (66.1%), 72 (12.1%) and 71 (11.9%) of the collected specimens of *Ae. aegypti* immatures, respectively (Additional file 2).

The seven water containers with *Ae. aegypti* immatures in the public area harbored a total of 283 specimens. Plastic tarps on the ground (138 specimens; 48.7%), stream/ditch (puddles) (99 specimens; 35%) and water tanks not connected to the water supply service (19 specimens; 6.7%) comprised most of the *Ae. aegypti* immature specimens found in the public area during the four surveys (Additional file 3).

### Household indices of *Ae. aegypti* eggs and adults

During the four survey cycles, 373 ovitraps were installed and recovered intact in the private area. Of them, 157 were positive for the presence of eggs (OPI: 42.1%), and 7299 eggs were counted on the pallets (EDI: 46.5 eggs). Indoor aspirations were also performed in 446 households, and 138 adults of *Ae. aegypti* mosquitoes were captured in 81 (18.2%) households (Table 1).

### Detection of non-*Ae. aegypti* species

No immature non-*Ae. aegypti* mosquitoes were found in the private area during any of the four survey cycles. However, immature *Culex quinquefasciatus* was found in the public space (2 in the second survey cycle and 31 in the third cycle). All 33 (100%) *Cx. quinquefasciatus* immatures collected in the public area were caught in standing water in streams or ditches. Also, during the indoor aspirations, 101 adult specimens of *Cx. quinquefasciatus* were captured in 55 (12.3%) of the 446 aspirations. Adult *Cx. quinquefasciatus* were aspirated in all four survey cycles.

### Temporal variation of *Ae. aegypti* entomological indices during the four cycles

**Fig. 2:**
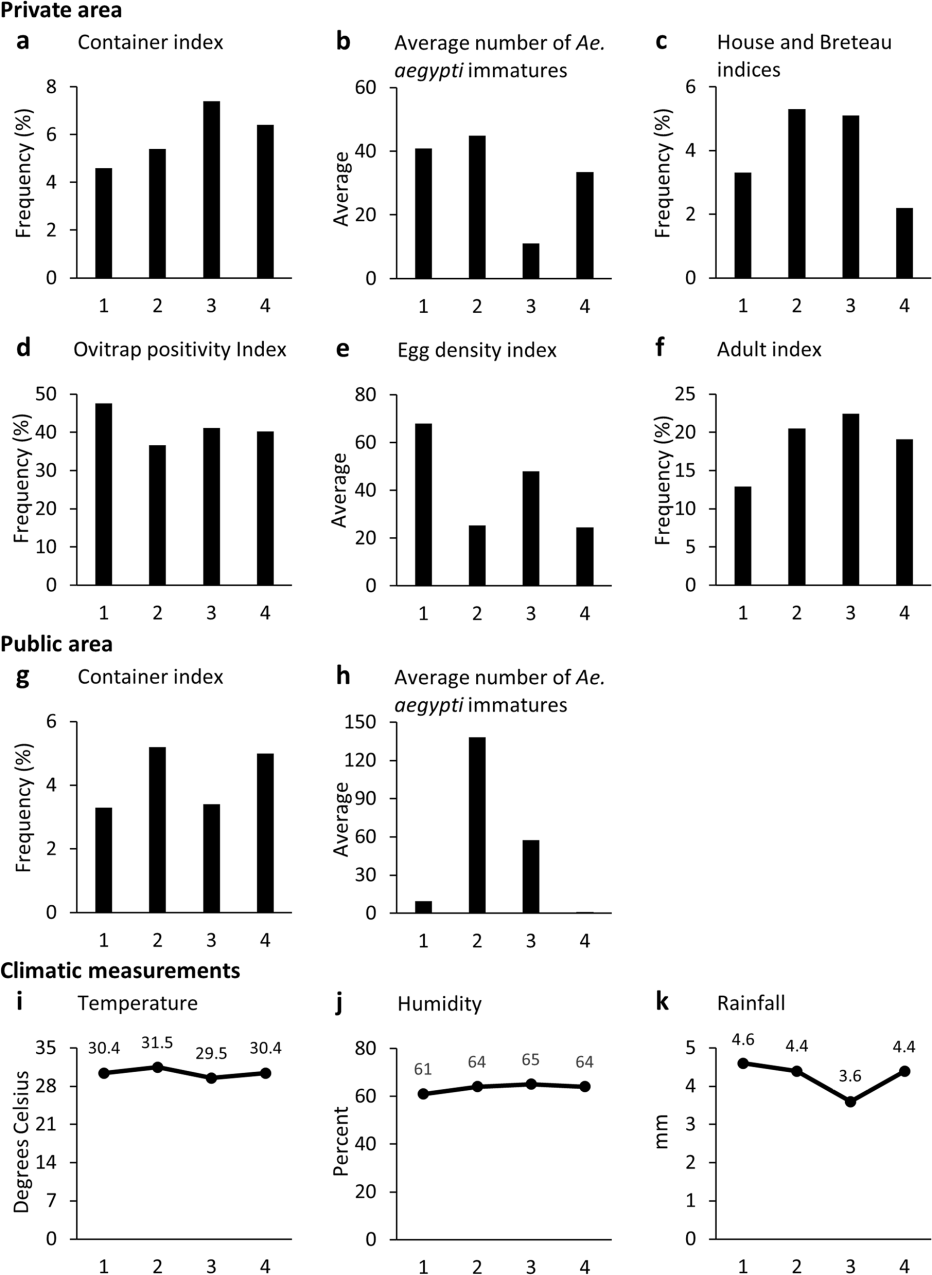
Entomological indices and climatic measurements for the four survey cycles. Private area: **a** Container index (frequency of potential breeding sites positive for *Aedes aegypti* immatures). **b** Average number of *Ae. aegypti* immatures per positive breeding site **c** House index (frequency of households with immatures in container) and Breteau Index (frequency of container with immatures per inspected households). **d** Ovitrap Positivity Index (frequency of positive ovitraps). **e** Egg Density Index (average number of eggs per positive trap). **f** Adult Index (frequency of household with adults indoor). Public area: **g** Container index. **h** Average number of *Ae. aegypti* immatures per positive breeding site. Climatic measurements: **i** Temperature (average values of the collection days). **j** Humidity (average values of the collection days). **k** Rainfall (average daily values for the period of the cycle). Survey cycle 1: September–December, 2019; Survey cycle 2: January–April, 2020; Survey cycle 3: September–December 2020; Survey cycle 4: January–April 2021

Table 1 and Fig. 2 show the measured entomological indices for each survey cycle. While some of the indices (CI, HI/BI) were relatively similar throughout the four surveys, others were not (frequency of potential breeding sites per surveyed household, average number of *Ae. aegypti* immatures per positive breeding site, OPI, EDI, and AI). In addition, no consistent pattern was observed among the indices during the four surveys or between the two pairs of surveys performed during the same period of different years (cycles 1 and 3 and cycles 2 and 4). Furthermore, no substantial variation in average daily temperature (range: 29.5 °C to 31.5 °C), humidity (61% to 65%) and rainfall levels (3.6 mm to 4.6 mm) was observed during the four survey cycles (Fig. 2) that could potentially explain the differences in entomological indices among the cycles. However, the rainfall levels were lower during the third survey cycle (Fig. 2k), and, in this same cycle, the average number of *Ae. aegypti* immatures per positive breeding site was substantially lower compared to the other cycles (Table 1, Fig. 2b).

### Spatial distribution of *Ae. aegypti* entomological indices

**Fig. 3:**
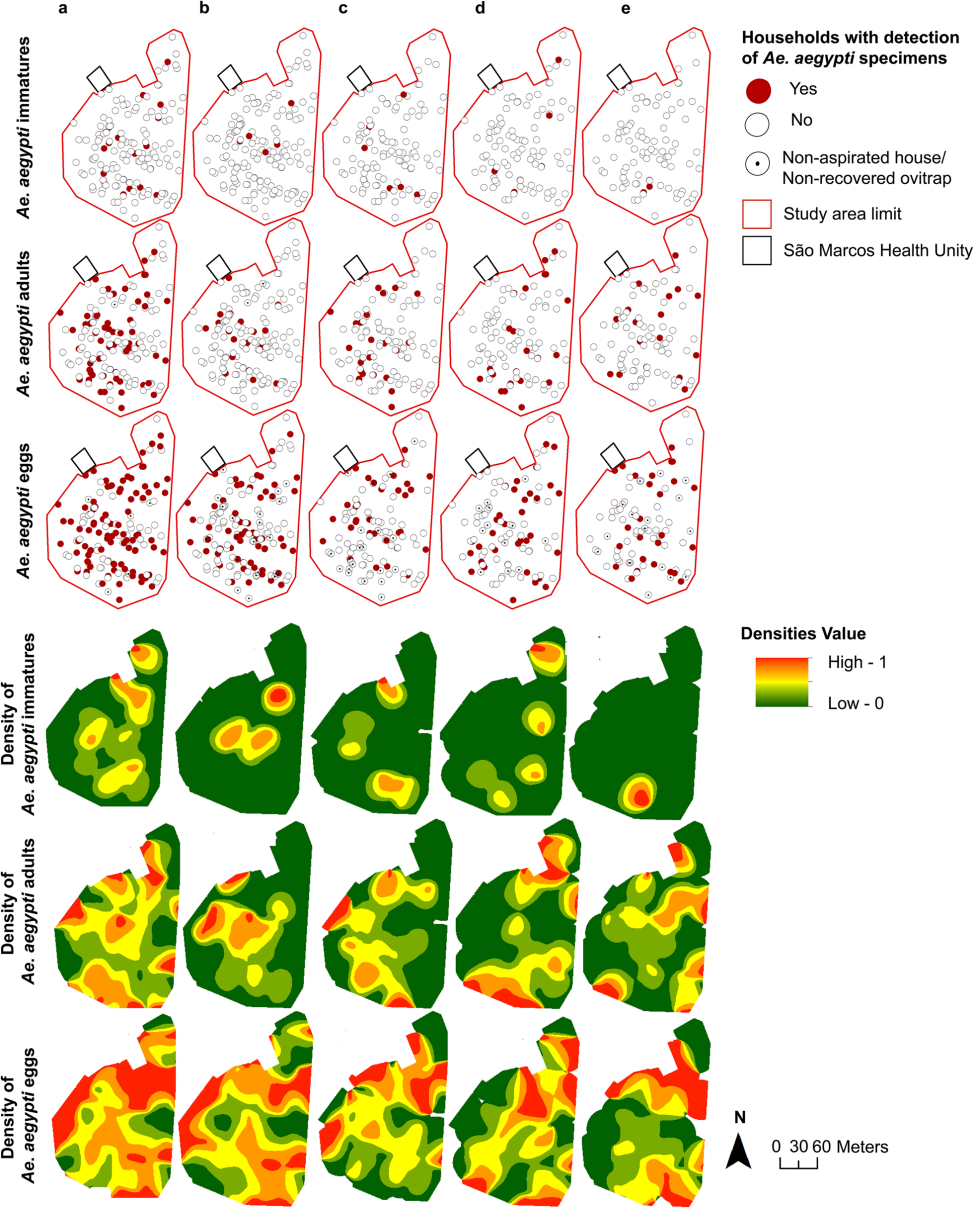
Spatial distribution of study households according to the collection of *Aedes aegypti* immatures, adults and eggs and respective kernel density-ratio maps overall and for each survey cycle, Pau da Lima neighborhood, Salvador, Brazil. **a** Overall (four cycles) **b** Survey cycle 1: September-December, 2019.** c** Survey cycle 2: January–April, 2020. **d** Survey cycle 3: September-December 2020. **e** Survey cycle 4: January–April 2021

Figure 3 shows the spatial distribution of the presence of eggs, immature and adult forms of *Ae. aegypti* for the surveyed households for the overall study period and each survey cycle separately, as well as their respective kernel density-ratio maps. The visual analysis of the maps did not reveal any hotspot that was detected consistently by more than one of the entomological markers within each survey cycle or overall. It also did not depict any clustering of a specific indicator between the cycles. Furthermore, the local Moran’s I analysis for spatial autocorrelation did not show any spatial clustering for the different *Ae. aegypti* forms.

### Factors associated with detection of *Ae. aegypti* immatures, eggs and adults

Considering the four survey cycles in the private area, immature *Ae. aegypti* was found more frequently in containers without coverage/protection (11.0%) compared to those with cover/protection (3.5%) (*P* < 0.01), in containers surrounded by plants or trees (14.7%) compared to those that were not (5.0%) (*P* = 0.02) and in containers whose water had visual evidence of organic matter (26.8%) compared to those without (3.6%) (*P* < 0.01) (Table 2). Immatures were also found more often in containers located outdoors (6.5%) compared to those located indoors (3.4%) (*P* = 0.41), but this difference was not statistically significant.

**Table 2:** Overall result for the four survey cycles of the frequency of *Ae. aegypti* immatures collected in breeding sites (water containers) located in private and public areas, according to observed characteristics of the breeding sites, Pau da Lima neighborhood, Salvador, Brazil, September 2019 to April 2021

Characteristic of the potential breeding site	No. of potential breeding sites	No. with *Aedes aegypti*	Frequency of Positive (%)	*P* value
Private area				
Location				
Outdoor	228	15	6.5	0.41
Indoor	88	3	3.4	
Cover/protection				
Yes	225	8	3.5	** < 0.01**
No	91	10	11.0	
Presence of plants or trees around^a^				
Yes	34	5	14.7	**0.02**
No	258	13	5.0	
Organic matter in the water^a^				
Yes	41	11	26.8	** < 0.01**
No	195	7	3.6	
Public area				
Cover/protection				
Yes	2	0	0.0	1.00
No	184	7	3.8	
Presence of plants or trees around				
Yes	55	0	0.0	0.10
No	131	7	5.3	
Organic matter in the water^a^				
Yes	75	4	5.3	0.44
No	109	3	2.7	

^a^These characteristics could not be evaluated in all the structures or reservoirs containing water

In contrast, using the data from the four surveys in the public area, no significant differences in the frequency of immature *Ae. aegypti* were observed regardless of whether breeding sites were covered/protected or not (0.0% vs. 3.8%, respectively, *P* = 1.00), whether there were plants or trees around them or not (0.0% vs. 5.3%, respectively, *P* = 0.10) or whether organic matter was present in the water or not (5.3% vs. 2.7%, respectively, *P* = 0.44) (Table 2).

Whether specific household characteristics were associated with the detection of *Ae. aegypti* eggs, immatures or adults was also investigated. The only statistically significant association found was between the presence of containers for water storage in the household and the detection of *Ae. aegypti* immatures (6.5% of the households with containers for water storage had immatures detected vs. 1.7% of the households without them, *P* = 0.01) (Table 3).

**Table 3:** Overall result for the four survey cycles of the frequency of *Ae. aegypti* indicators (eggs, immatures and adults), according to the socio-environmental characteristics of the household, Pau da Lima neighborhood, Salvador, Brazil, September 2019 to April 2021

Socio-environmental characteristics of the households	Number of households		% of positive (%)	*P* value	Number of households		% of positive (%)	*P* value	Number of households		% of Positive (%)	*P* value
	Inspected	With *Ae. aegypti* immature			With recovered ovitrap	With *Ae. aegypti* egg			Aspirated	With *Ae. aegypti* adult		
Irregular water provision												
Yes	240	11	4.5	0.51	201	81	40.2	0.44	238	65	27.3	0.43
No	208	7	3.3		172	76	44.1		208	50	24.0	
Water storage containers (water reservoirs)												
Yes	215	14	6.5	**0.01**	183	72	39.3	0.29	214	59	27.5	0.40
No	233	4	1.7		190	85	44.7		232	56	24.1	
Floor level												
Ground	341	12	3.5	0.33	283	125	44.1	0.14	339	92	27.1	0.24
First or second	107	6	5.6		90	32	35.5		107	23	21.4	
Residence located on an unpaved street												
Yes	4	0	0.0	1.00	4	1	25.0	0.64	4	1	25.0	1.00
No	444	18	4.0		369	156	42.2		442	114	25.7	
Type of residence construction												
Plastered walls	442	17	3.8	0.21	369	154	41.7	0.31	441	115	26.0	0.33
Unplastered walls	6	1	16.6		4	3	75.0		5	0	0.0	
No. of inhabitants												
≤ 5	414	17	4.1	1.00	345	149	43.1	0.13	413	104	25.1	0.30
> 5	34	1	2.9		28	8	28.5		33	11	33.3	

The total number of households for each of the evaluated outcomes (eggs, immatures or adults) varies because we only analyzed households that had ovitraps recovered, whose breeding sites were accessible for evaluation of immatures or that were aspirated

### Investigation of arbovirus RNA in the pools of adult *Ae. aegypti*

The 64 pools of adult female specimens of *Ae. aegypti* (34 pools of engorged and 30 pools of non-engorged mosquitoes) were tested by qRT-PCR for DENV, ZIKV and CHIKV. The pool size ranged from one to three mosquitoes (median = 1). All tests were negative for the three arboviruses.

## Discussion

This series of entomological surveys in a low-income urban community in Brazil confirms that *Ae. aegypti* reproduction occurs in both households and public spaces. However, although the relative frequency in which immatures of *Ae. aegypti* were found in water containers located in the private area was similar to that found in the public space (5.7% and 3.7%, respectively), the absolute number of potential breeding sites (structures or reservoirs containing water) in the private area was much higher compared to the public area (316 versus 186, respectively), as was the total number of breeding sites containing immature *Ae. aegypti* (18 versus 7, respectively). These differences are likely to have been much larger had we surveyed all households in the study site rather than a sample. Despite the relatively more significant role of the household environment in *Ae. aegypti* reproduction, integrated actions to reduce vector infestation need to target both spaces.

In the private areas, water buckets and tanks were the most frequent types of water containers and the ones that contributed most to the growth of *Ae. aegypti* immatures. Notably, in 70% of the survey inspections, a potential breeding site was found in the households; most often, they were a type of water reservoir used to store water for daily needs. Furthermore, the presence of a water storage reservoir was significantly associated with detecting immatures in the house, reinforcing that containers used to accumulate water were the mainstay for *Ae. aegypti* proliferation in this community [5, 26–28]. A regular supply of potable water to households is thus a pivotal action to reduce the population’s need for water storage, potentially reducing *Ae. aegypti* reproduction and arboviral transmission risk.

Although not statistically significant, the frequency of immatures between water sources located inside the households was almost double the frequency outside. Other studies have reported similar findings, which may have important implications for vector control practices [5, 6, 26, 27]. As expected, the frequency of immatures in covered water sources was much lower than in uncovered ones. Thus, given the observed need of low-income urban communities to store water, intersectoral actions by the health, infrastructure and education authorities to instruct the population regarding the importance of adequately closing their water tanks, as well as arrange for the provision of covered water tanks to those who can't afford them, are needed [28]. In contrast, most mosquito breeding sites in the public area comprised water accumulated in the urban environment that cannot be covered, such as puddled water or abandoned containers and garbage. Thus, the interventions to mitigate *Ae. aegypti* reproduction in the public space must be based on sanitation measures, including improving the water drainage system and solid waste management.

Other container characteristics can also influence the capacity of the accumulated water to serve as a breeding site [29]. In this study, the presence of organic matter in the water favored the detection of *Ae. aegypti* immatures, especially in the private area, where the association was statistically significant. Although *Culex* mosquitoes typically reproduce in water rich in organic matter [19], our finding confirms that *Ae. aegypti* has also adapted to reproducing in non-traditional breeding sites rich in organic matter, such as puddles, rainwater drainage structures and sewers [14–16, 28]. This study found that the presence of vegetation surrounding the water containers largely increased the positivity of the breeding site for immatures in the private area. Proximity to vegetation might affect container positivity as vegetation is a vital sugar-feeding resource and serves as a resting site for adult mosquitoes [29].

Climatic factors in Salvador do not vary much. Average temperatures oscillate by 2.8 °C throughout the year, and rainfall occurs in all months, with December being the wettest month (average of 189 mm) and May the driest (average of 51 mm) [30]. In places where the climatic factors present more significant variation, entomological index fluctuation may be more evident, often showing an increase in *Ae. aegypti* infestation in higher temperatures and lower precipitation [26, 29, 31]. The absence of a trend in *Ae. aegypti* infestation indices measured during the four survey cycles may reflect the limited fluctuations in Salvador’s climatic conditions, but the short series of surveys preclude a definitive conclusion. Nevertheless, during the third survey cycle, both rainfall levels and the average number of *Ae. aegypti* immatures per positive breeding site were the lowest. This may reflect a potential association between less available water and reduced abundance of immatures in the environment. However, other entomological indices did not show reduced levels in the same cycle.

In the private area, a substantial decrease in the frequency of potential breeding sites during the last survey cycle was detected (Table 1). This reduction may reflect the researchers’ guidance to residents to avoid inadequate water accumulation at each household visit, or it may simply result from temporal fluctuation. Despite the variation in the frequency in which potential breeding sites were found, the frequency of breeding sites with *Ae. aegypti* immatures did not vary significantly during the four cycles. Further investigations in low-income urban communities are needed to elucidate whether there is any relationship between the availability of reservoirs with accumulated water and the frequency in which potential breeding sites harbor *Ae. aegypti* immatures.

Regarding the *Ae. aegypti* entomological indicators, there is no consensus on which of them (eggs or immatures) would be more adequate to predict the presence of the adult mosquito, the stage directly linked to arbovirus transmission [32]. Manrique-Saide et al. showed an association between outdoor ovitrap and indoor adult positivity, suggesting that ovitrap collections may represent a practical method of monitoring the presence of indoor *Ae. aegypti* females [32]. They and other authors have shown that surveys based on oviposition traps may be more sensitive than indoor adult mosquito collection [32–34]. In this study, the OPI was approximately twice as high as the AI for each cycle. Compared with the other entomological indices, the OPI was the most representative in all cycles (Table 1). If oviposition traps are indeed the most sensitive method to predict the presence of adult mosquitoes, the reliance of vector control programs on traditional indices based on *Ae. aegypti* larvae or pupae as indicators of adult infestations needs to be revisited.

In this study, no consistent pattern was found in the spatial distribution of the *Ae. aegypti* eggs, immatures or adults, within each survey and over time. This may be explained by the fact that our surveys were carried out in a small area in relation to the dispersal capacity of *Ae. aegypti* and with relatively similar social and environmental characteristics. In other studies, performed over larger areas, researchers demonstrated that *Ae. aegypti* distribution is highly focal and that hotspots of high vector abundance at the level of small groups of houses are common but temporally unstable. Thus, hotspots observed during one survey did not necessarily predict hotspots at the same location during subsequent surveys, which imposes a significant challenge to intervention strategies targeting vector control on highly infested locations [35, 36]. In addition, the discordance that we observed between the areas where eggs, immatures and adults were found may suggest that when used separately, none of these entomological indicators is accurate enough to capture the spatial distribution of *Ae. aegypti* in a low-income urban area.

Although pools of mosquitoes positive for DENV, ZIKV and CHIKV were not found, Salvador has been an epicenter for epidemics of these arboviruses [1, 37, 38]. Detecting arbovirus in mosquitoes is challenging during non-epidemic periods, especially when the presence of human-infected cases in the locality does not guide mosquito captures. This may explain the negative results in the tested pools. In Salvador, CHIKV transmission was ongoing between the first and second surveys, and it exploded between the second and third survey cycles. However, an additional survey between the second and third cycles of surveys could not be performed because of the COVID-19 pandemic isolation recommendations [39]. Furthermore, during the whole study period, the transmission of DENV and ZIKV in Salvador was very low [40, 41]. Finally, a relatively small number of mosquitoes was tested, reducing the chance of arbovirus detection. Our study has other limitations. The relatively short study period could have hindered the identification of temporal patterns regarding the *Ae. aegypti* indices. Moreover, 60 (40%) households were lost to follow-up during the four survey cycles, and not all installed ovitraps were successfully recovered. These two factors may have also limited the power of our analysis.

## Conclusion

The study findings highlight the versatility of *Ae. aegypti* mosquitoes, which can reproduce in various habitats in low-income urban communities, challenging the prevention of arbovirus transmission in such settings. Notably, even within this relatively small community, a high degree of heterogeneity across space and time regarding mosquito presence and abundance was observed. This may suggest that *Ae. aegypti* reproduction is widespread in low-income urban communities, likely switching locations in response to favorable or hostile conditions for mosquito breeding and resting. Therefore, focal interventions to reduce the vector in an area considered propitious to *Aedes* reproduction may have limited effectiveness if the surroundings are not targeted as well, because the vector may find other suitable habitats nearby.

Furthermore, the discrepancy in findings based on the different mosquito collection methods highlights the limitations of many previous studies, which often relied on only one or two methods. As no single entomological survey method is sufficiently representative, mosquito surveillance systems should be comprehensive, including a range of approaches to collect vectors in their different life stages. Finally, the finding that household storage of water, as well as uncovered accumulation of water in households, ditches and solid waste disposals, is critical for *Ae. aegypti* proliferation indicates that efforts should be made to improve basic sanitation services in low-income settlements, especially the provision of regular water supply, rainwater drainage and solid waste management. Further long-term studies in larger low-income communities are needed to confirm our findings on vector spatial density distribution and their underlying predisposing conditions.

## Supplementary Information

**Additional file 1.** Study area and surveyed households in each of the four survey cycles, Pau da Lima neighborhood, Salvador, Brazil. a Survey cycle 1: September–December 2019. b Survey cycle 2: January–April 2020. c Survey cycle 3: September–December 2020. d Survey cycle 4: January–April 2021.

**Additional file 2.** Frequency of Aedes aegypti immatures collected in breeding siteslocated in the surveyed households during the four cycles of entomological surveys performed at the Pau da Lima neighborhood, Salvador, Brazil.

**Additional file 3.** Frequency of Aedes aegypti immatures collected in breeding siteslocated in the public area during the four cycles of entomological surveys performed at the Pau da Lima neighborhood, Salvador, Brazil.

## Data Availability

The datasets used and/or analyzed during the current study are available from the corresponding author upon reasonable request.

## Abbreviations

DENV: Dengue virus
ZIKV: Zika virus
CHIKV: Chikungunya virus
RT-PCR: Real-time polymerase chain reaction
REDCAP: Research electronic data capture
CI: Container Index
OPI: Ovitrap Positivity Index
EDI: Egg Density Index
HI: House Index
BI: Breteau Index
AI: Adults Index
