# Supplementary figures and images for: Density of Aedes aegypti (Diptera: Culicidae) in a low-income Brazilian urban community where dengue, Zika, and chikungunya viruses co-circulate
Source: Parasit Vectors. 2023 May 6;16:159. doi: 10.1186/s13071-023-05766-5 (PMC10163576; doi:10.1186/s13071-023-05766-5)

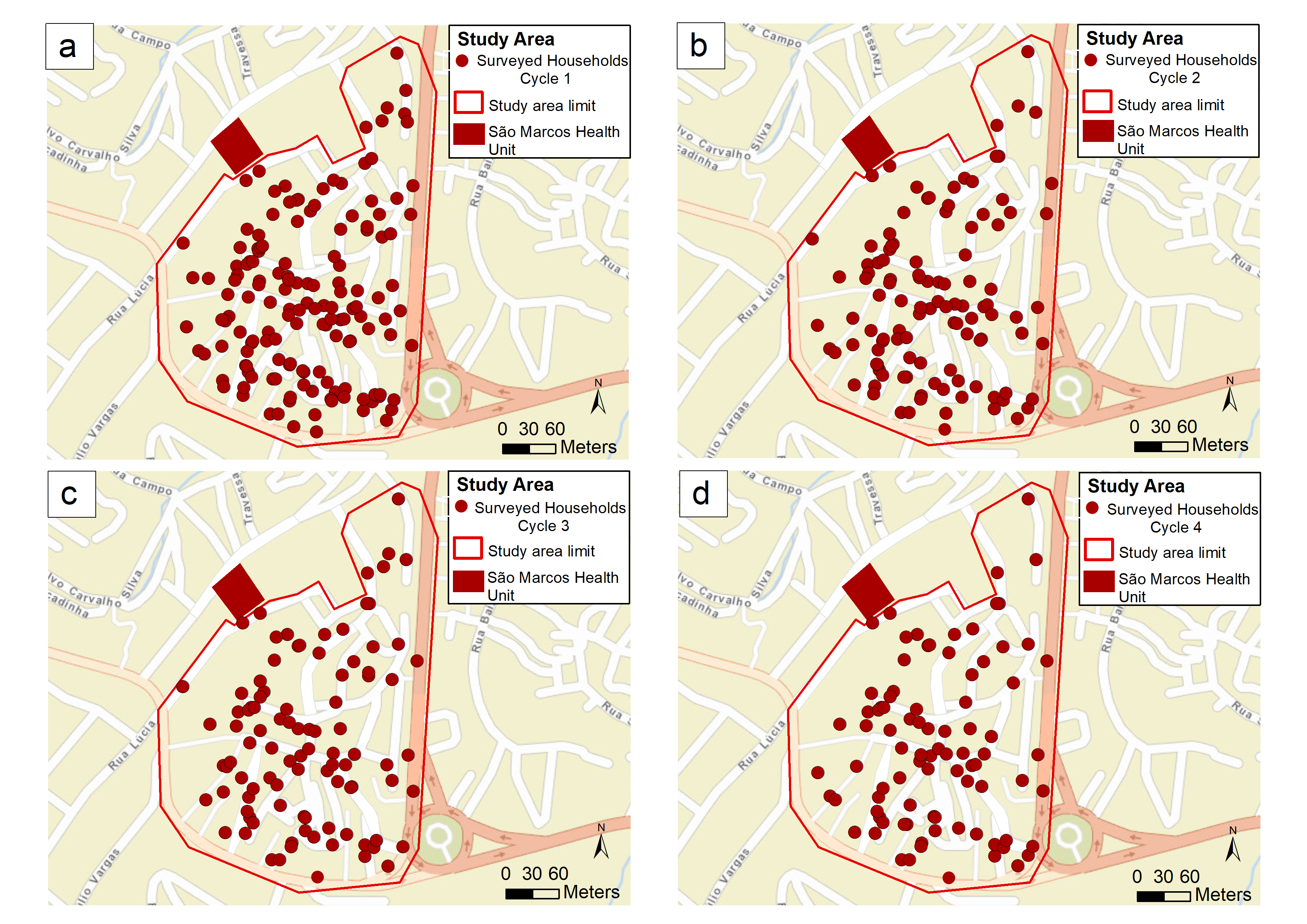

Supplement: Supplementary file 1 — Additional file 1. Study area and surveyed households in each of the four survey cycles, Pau da Lima neighborhood, Salvador, Brazil. a Survey cycle 1: September–December 2019. b Survey cycle 2: January–April 2020. c Survey cycle 3: September–December 2020. d Survey cycle 4: January–April 2021. [file 13071_2023_5766_MOESM1_ESM.tif]
