# Supplementary material for: Density of Aedes aegypti (Diptera: Culicidae) in a low-income Brazilian urban community where dengue, Zika, and chikungunya viruses co-circulate
Source: Parasit Vectors. 2023 May 6;16:159. doi: 10.1186/s13071-023-05766-5 (PMC10163576; doi:10.1186/s13071-023-05766-5)
Supplement: Supplementary file 2 — Additional file 2. Frequency of Aedes aegypti immatures collected in breeding siteslocated in the surveyed households during the four cycles of entomological surveys performed at the Pau da Lima neighborhood, Salvador, Brazil. [file 13071_2023_5766_MOESM2_ESM.docx]

**Additional file 2.** Frequency of *Ae. aegypti* immatures collected in breeding sites (water containers) located in the surveyed households during the four cycles of entomological surveys performed at the Pau da Lima neighborhood, Salvador, Brazil.

| **Type of water container identified during the surveys** | **Cycle 1** | | | **Cycle 2** | | | **Cycle 3** | | | **Cycle 4** | | | **Total** | | |
| --- | --- | --- | --- | --- | --- | --- | --- | --- | --- | --- | --- | --- | --- | --- | --- |
|  | **No. of containers (%)** | **No. with *Ae. aegypti* (%)** | **No. of *Ae. aegypti* specimens** | **No. of containers (%)** | **No. with *Ae. aegypti* (%)** | **No. of *Ae. aegypti* specimens** | **No. of containers (%)** | **No. with *Ae. aegypti* (%)** | **No. of *Ae. aegypti* specimens** | **No. of containers (%)** | **No. with *Ae. aegypti* (%)** | **No. of *Ae. aegypti* specimens** | **No. of containers (%)** | **No. with *Ae. aegypti* (%)** | **No. of *Ae. aegypti* specimens** |
| Water bucket | 41 (38.4) | 2 (4.8) | 144 | 49 (44.1) | 3 (6.1) | 249 | 20 (29.9) | 0 (0.0) | 0 | 4 (12.9) | 0 (0.0) | 0 | 114 (36.1) | 5 (4.3) | 393 |
| Water tank, connected to the water supply service | 35 (32.8) | 0 (0.0) | 0 | 19 (17.2) | 0 (0.0) | 0 | 28 (41.8) | 1 (3.5) | 5 | 21 (67.8) | 2 (9.5) | 67 | 103 (32.6) | 3 (2.9) | 72 |
| Watter vat | 10 (9.4) | 1 (10.0) | 1 | 12 (10.8) | 0 (0.0) | 0 | 5 (7.4) | 0 (0.0) | 0 | 1 (3.2) | 0 (0.0) | 0 | 28 (8.9) | 1 (3.5) | 1 |
| Water tank, not connected to the water supply service | 8 (7.4) | 0 (0.0) | 0 | 9 (8.1) | 2 (22.2) | 6 | 8 (11.9) | 1 (12.5) | 27 | 5 (16.1) | 0 (0.0) | 0 | 30 (9.5) | 3 (10) | 33 |
| Plastic container | 7 (6.5) | 1 (14.2) | 57 | 11 (9.9) | 1 (9) | 14 | 0 (0.0) | 0 (0.0) | 0 | 0 (0.0) | 0 (0.0) | 0 | 18 (5.7) | 2 (11.1) | 71 |
| Plant vase | 2 (1.9) | 1 (50.0) | 2 | 1 (0.9) | 0 (0.0) | 0 | 1 (1.5) | 0 (0.0) | 0 | 0 (0.0) | 0 (0.0) | 0 | 4 (1.3) | 1 (25) | 2 |
| Cistern | 1 (0.9) | 0 (0.0) | 0 | 1 (0.9) | 0 (0.0) | 0 | 1 (1.5) | 1 (100) | 15 | 0 (0.0) | 0 (0.0) | 0 | 3 (1) | 1 (33.3) | 15 |
| Bottle / can | 1 (0.9) | 0 (0.0) | 0 | 2 (1.8) | 0 (0.0) | 0 | 0 (0.0) | 0 (0.0) | 0 | 0 (0.0) | 0 (0.0) | 0 | 3 (1) | 0 (0.0) | 0 |
| Religious object | 0 (0.0) | 0 (0.0) | 0 | 1 (0.9) | 0 (0.0) | 0 | 1 (1.5) | 1 (100) | 4 | 0 (0.0) | 0 (0.0) | 0 | 2 (0.6) | 1 (50) | 4 |
| Sink | 1 (0.9) | 0 (0.0) | 0 | 1 (0.9) | 0 (0.0) | 0 | 0 (0.0) | 0 (0.0) | 0 | 0 (0.0) | 0 (0.0) | 0 | 2 (0.6) | 0 (0.0) | 0 |
| Home drain | 0 (0.0) | 0 (0.0) | 0 | 1 (0.9) | 0 (0.0) | 0 | 1 (1.5) | 0 (0.0) | 0 | 0 (0.0) | 0 (0.0) | 0 | 2 (0.6) | 0 (0.0) | 0 |
| Toilet tank | 0 (0.0) | 0 (0.0) | 0 | 2 (1.8) | 0 (0.0) | 0 | 0 (0.0) | 0 (0.0) | 0 | 0 (0.0) | 0 (0.0) | 0 | 2 (0.6) | 0 (0.0) | 0 |
| Pan/cauldron | 1 (0.9) | 0 (0.0) | 0 | 1 (0.9) | 0 (0.0) | 0 | 0 (0.0) | 0 (0.0) | 0 | 0 (0.0) | 0 (0.0) | 0 | 2 (0.6) | 0 (0.0) | 0 |
| Broken fan | 0 (0.0) | 0 (0.0) | 0 | 1 (0.9) | 0 (0.0) | 0 | 0 (0.0) | 0 (0.0) | 0 | 0 (0.0) | 0 (0.0) | 0 | 1 (0.3) | 0 (0.0) | 0 |
| Concrete block | 0 (0.0) | 0 (0.0) | 0 | 0 (0.0) | 0 (0.0) | 0 | 1 (1.5) | 1(100) | 4 | 0 (0.0) | 0 (0.0) | 0 | 1 (0.3) | 1(100) | 4 |
| Gallon water | 0 (0.0) | 0 (0.0) | 0 | 0 (0.0) | 0 (0.0) | 0 | 1 (1.5) | 0 (0.0) | 0 | 0 (0.0) | 0 (0.0) | 0 | 1 (0.3) | 0 (0.0) | 0 |
| **Total** | **107 (100)** | **5 (4.6)** | **204** | **111 (100)** | **6 (5.4)** | **269** | **67 (100)** | **5 (7.4)** | **55** | **31 (100)** | **2 (6.4)** | **67** | **316 (100)** | **18 (5.7)** | **595** |

**Note:** Survey cycle 1: September-December, 2019; Survey cycle 2: January-April, 2020; Survey cycle 3: September-December 2020; Survey cycle 4: January-April 2021.
