# Supplementary material for: Density of Aedes aegypti (Diptera: Culicidae) in a low-income Brazilian urban community where dengue, Zika, and chikungunya viruses co-circulate
Source: Parasit Vectors. 2023 May 6;16:159. doi: 10.1186/s13071-023-05766-5 (PMC10163576; doi:10.1186/s13071-023-05766-5)
Supplement: Supplementary file 3 — Additional file 3. Frequency of Aedes aegypti immatures collected in breeding siteslocated in the public area during the four cycles of entomological surveys performed at the Pau da Lima neighborhood, Salvador, Brazil. [file 13071_2023_5766_MOESM3_ESM.docx]

**Additional file 3.** Frequency of *Ae. aegypti* immatures collected in breeding sites (water containers) located in the public area during the four cycles of entomological surveys performed at the Pau da Lima neighborhood, Salvador, Brazil.

|  | **Cycle 1** | | | **Cycle 2** | | | **Cycle 3** | | | | **Cycle 4** | | | **Total** | | |
| --- | --- | --- | --- | --- | --- | --- | --- | --- | --- | --- | --- | --- | --- | --- | --- | --- |
| **Type of water container identified during the surveys** | **No. of containers (%)** | **No. with**  ***Ae. aegypti* (%)** | **No. of *Ae. aegypti***  **specimens** | **No. of containers (%)** | **No. with**  ***Ae. aegypti* (%)** | **No. of *Ae. aegypti***  **specimens** | **No. of containers (%)** | **No. with**  ***Ae. aegypti* (%)** | **No. of *Ae. aegypti* specimens** | | **No. of containers (%)** | **No. with *Ae. aegypti* (%)** | **No. of *Ae. aegypti* specimens** | **No. of containers (%)** | **No. with *Ae. aegypti* (%)** | **No. of *Ae. aegypti* specimens** |
|  |  |  |  |  |  |  |  |  |  |  |  |  |  |  |  |  |
| Plastic container | 46 (51.7) | 0 (0.0) | 0 | 4 (21) | 0 (0.0) | 0 | 32 (55.2) | 1 (3.1) | | 17 | 5 (25) | 0 (0.0) | 0 | 87 (46.8) | 1 (1.1) | 17 |
| Stream / ditch (puddled) | 16 (18) | 0 (0.0) | 0 | 6 (31.6) | 0 (0.0) | 0 | 12 (20.7) | 1 (8.3) | | 98 | 10 (50) | 1 (10) | 1 | 44 (23.6) | 2 (4.5) | 99 |
| Puddle | 10 (11.2) | 0 (0.0) | 0 | 5 (26.4) | 0 (0.0) | 0 | 5 (8.7) | 0 (0.0) | | 0 | 3 (15) | 0 (0.0) | 0 | 23 (12.3) | 0 (0.0) | 0 |
| Plastic bag | 5 (5.8) | 0 (0.0) | 0 | 0 (0.0) | 0 (0.0) | 0 | 1 (1.7) | 0 (0.0) | | 0 | 0 (0.0) | 0 (0.0) | 0 | 6 (3.1) | 0 (0.0) | 0 |
| Water bucket | 3 (3.4) | 0 (0.0) | 0 | 1(5.2) | 0 (0.0) | 0 | 1 (1.7) | 0 (0.0) | | 0 | 0 (0.0) | 0 (0.0) | 0 | 5 (2.6) | 0 (0.0) | 0 |
| Bottle / can | 0 (0.0) | 0 (0.0) | 0 | 0 (0.0) | 0 (0.0) | 0 | 3 (5.2) | 0 (0.0) | | 0 | 1 (5) | 0 (0.0) | 0 | 4 (2.1) | 0 (0.0) | 0 |
| Styrofoam box | 1 (1.1) | 0 (0.0) | 0 | 1(5.2) | 0 (0.0) | 0 | 1 (1.7) | 0 (0.0) | | 0 | 1 (5) | 0 (0.0) | 0 | 4 (2.1) | 0 (0.0) | 0 |
| Toilet (unused) | 1 (1.1) | 1 (100) | 5 | 0 (0.0) | 0 (0.0) | 0 | 1 (1.7) | 0 (0.0) | | 0 | 0 (0.0) | 0 (0.0) | 0 | 2 (1) | 1 (50) | 5 |
| Plastic tarp on the ground | 0 (0.0) | 0 (0.0) | 0 | 2 (10.6) | 1 (50) | 138 | 0 (0.0) | 0 (0.0) | | 0 | 0 (0.0) | 0 (0.0) | 0 | 2 (1) | 1 (50) | 138 |
| Blender cup | 1 (1.1) | 0 (0.0) | 0 | 0 (0.0) | 0 (0.0) | 0 | 0 (0.0) | 0 (0.0) | | 0 | 0 (0.0) | 0 (0.0) | 0 | 1 (0.6) | 0 (0.0) | 0 |
| Construction debris | 1 (1.1) | 0 (0.0) | 0 | 0 (0.0) | 0 (0.0) | 0 | 0 (0.0) | 0 (0.0) | | 0 | 0 (0.0) | 0 (0.0) | 0 | 1 (0.6) | 0 (0.0) | 0 |
| Sewer | 1 (1.1) | 0 (0.0) | 0 | 0 (0.0) | 0 (0.0) | 0 | 0 (0.0) | 0 (0.0) | | 0 | 0 (0.0) | 0 (0.0) | 0 | 1 (0.6) | 0 (0.0) | 0 |
| Umbrella | 1 (1.1) | 0 (0.0) | 0 | 0 (0.0) | 0 (0.0) | 0 | 0 (0.0) | 0 (0.0) | | 0 | 0 (0.0) | 0 (0.0) | 0 | 1 (0.6) | 0 (0.0) | 0 |
| Metal cover | 1 (1.1) | 0 (0.0) | 0 | 0 (0.0) | 0 (0.0) | 0 | 0 (0.0) | 0 (0.0) | | 0 | 0 (0.0) | 0 (0.0) | 0 | 1 (0.6) | 0 (0.0) | 0 |
| Water tank, not connected to the water supply service | 1 (1.1) | 1 (100) | 19 | 0 (0.0) | 0 (0.0) | 0 | 0 (0.0) | 0 (0.0) | | 0 | 0 (0.0) | 0 (0.0) | 0 | 1 (0.6) | 1 (100) | 19 |
| Washing machine (unused) | 1 (1.1) | 1 (100) | 5 | 0 (0.0) | 0 (0.0) | 0 | 0 (0.0) | 0 (0.0) | | 0 | 0 (0.0) | 0 (0.0) | 0 | 1 (0.6) | 1 (100) | 5 |
| Sink plunger | 0 (0.0) | 0 (0.0) | 0 | 0 (0.0) | 0 (0.0) | 0 | 1 (1.7) | 0 (0.0) | | 0 | 0 (0.0) | 0 (0.0) | 0 | 1 (0.6) | 0 (0.0) | 0 |
| Plastic pool (abandoned) | 0 (0.0) | 0 (0.0) | 0 | 0 (0.0) | 0 (0.0) | 0 | 1 (1.7) | 0 (0.0) | | 0 | 0 (0.0) | 0 (0.0) | 0 | 1 (0.6) | 0 (0.0) | 0 |
| **Total** | **89 (100)** | **3 (3.3)** | **29** | **19 (100)** | **1 (5.2)** | **138** | **58 (100)** | **2 (3.4)** | | **115** | **20 (100)** | **1 (5)** | **1** | **186 (100)** | **7 (3.7)** | **283** |

**Note:** Survey cycle 1: September-December, 2019; Survey cycle 2: January-April, 2020; Survey cycle 3: September-December 2020; Survey cycle 4: January-April 2021.
